# Modeling Traumatic Brain Injury in Human Cerebral Organoids

**DOI:** 10.3390/cells10102683

**Published:** 2021-10-07

**Authors:** Santiago Ramirez, Abhisek Mukherjee, Sofia Sepulveda, Andrea Becerra-Calixto, Nicolas Bravo-Vasquez, Camila Gherardelli, Melissa Chavez, Claudio Soto

**Affiliations:** Mitchell Center for Alzheimer’s Disease and Related Brain Disorders, Department of Neurology, McGovern Medical School, University of Texas Health Science at Houston, Houston, TX 77030, USA; Santiago.D.Ramirez@uth.tmc.edu (S.R.); Abhisek.Mukherjee@uth.tmc.edu (A.M.); Sofia.E.SepulvedaContreras@uth.tmc.edu (S.S.); Andrea.d.BecerraCalixto@uth.tmc.edu (A.B.-C.); Nicolas.A.BravoVasquez@uth.tmc.edu (N.B.-V.); Camila.GherardelliBrooks@uth.tmc.edu (C.G.); Melissa.V.Chavez@uth.tmc.edu (M.C.)

**Keywords:** cerebral organoids, traumatic brain injury, disease modeling, Alzheimer’s disease, amyloid plaques, neurofibrillary tangles

## Abstract

Traumatic brain injury (TBI) is a head injury that disrupts the normal brain structure and function. TBI has been extensively studied using various in vitro and in vivo models. Most of the studies have been done with rodent models, which may respond differently to TBI than human nerve cells. Taking advantage of the recent development of cerebral organoids (COs) derived from human induced pluripotent stem cells (iPSCs), which resemble the architecture of specific human brain regions, here, we adapted the controlled cortical impact (CCI) model to induce TBI in human COs as a novel in vitro platform. To adapt the CCI procedure into COs, we have developed a phantom brain matrix, matching the mechanical characteristics of the brain, altogether with an empty mouse skull as a platform to allow the use of the stereotactic CCI equipment on COs. After the CCI procedure, COs were histologically prepared to evaluate neurons and astrocyte populations using the microtubule-associated protein 2 (MAP2) and the glial fibrillary acidic protein (GFAP). Moreover, a marker of metabolic response, the neuron-specific enolase (NSE), and cellular death using cleaved caspase 3 were also analyzed. Our results show that human COs recapitulate the primary pathological changes of TBI, including metabolic alterations related to neuronal damage, neuronal loss, and astrogliosis. This novel approach using human COs to model TBI in vitro holds great potential and opens new alternatives for understanding brain abnormalities produced by TBI, and for the development and testing of new therapeutic approaches.

## 1. Introduction

Traumatic brain injury (TBI) is a head injury caused by a blow, bump, or jolt to the head or body or a penetrating head injury, associated with accidents, contact sports, and military duties that result in disruption of normal brain structure and function [1,2,3]. Worldwide, TBI is a major cause of death and disability with an estimate of 10 million people affected annually, among whom many survive, but with lifelong disabilities [4]. The pathology of TBI is complex and multifactorial, with the damage commonly categorized into primary and secondary injuries [5,6]. The primary injury occurs simultaneously with the impact and depending on the severity may lead to structural damage, inflammation, axonal shear, and cell death, causing headache, contusion, hemorrhage, loss of consciousness, skull fractures, loss of cerebral mass, and even death [6]. The secondary injury evolves during an extended period and includes a cascade of metabolic, inflammatory, and degenerative changes [7], which may lead to several neurodegenerative diseases, including Chronic Traumatic Encephalopathy (CTE), Alzheimer’s disease (AD), and other forms of dementia or movement disorders [8,9,10,11].

To understand the complex cascade of biological events in TBI, several rodent models have been developed [12]. However, the mouse brain differs from a human brain in the complexity, proportion, and distribution of different brain areas and their gene expression profiles [13]. The rodents-based TBI models are very useful to reproduce some aspects of the disease pathology [14]. Nevertheless, given the extensive spatial and temporal involvement of different cell types and signaling networks in TBI pathology, it is crucial to model TBI abnormalities in human cells, in their spatial context, to generate an effective translational model. Stretch and shear-based in vitro culture systems have been developed to model TBI in neurons derived from human induced pluripotent stem cells (iPSCs) [15,16,17]. However, these in vitro platforms do not have the three-dimensional organization and complexity of the brain, nor the adequate extracellular matrix necessary to model the biophysical interactions after the mechanical damage. Recent technological advances enabled in vitro generation of 3D brain-like structures, called cerebral organoids (COs) [18] which hold great potential as in vitro model of the human brain biological and disease pathways [19]. These structures resemble the cellular composition and positional organization of different anatomical regions of the human brain [17], such as the midbrain, thalamus [20], and cerebral cortex [21,22,23]. Furthermore, COs closely mimic the pattern of gene expression and epigenetic signature of the human brain [24,25,26]. Brain organoids can be generated from iPSCs with over 90% reproducibility [23]. In fact, organoid-to-organoid variability is comparable to that of individual human brains [23]. COs recapitulate the species-specific features of the human brain [27]. Consequently, the pathological cascade of several brain diseases that specifically affect humans has been investigated in brain organoids, including microcephaly [22], Zika virus infection [28], and autism spectrum disorders [29]. We and others have recently modeled the key pathological features of Alzheimer’s disease (AD) in brain organoids [30,31,32]. Remarkably, we found that when COs were generated from IPSCs derived from patients affected by inherited AD (but not healthy controls), the organoids developed over time the main pathological features of AD: Aβ amyloid plaques, Tau neurofibrillary tangles, and neurodegeneration [30].

Here, we sought to develop a human COs-based model of TBI as an improved in vitro system to study TBI. For this purpose, we adapted for COs the Controlled Cortical Impact (CCI), one of the most established and widely used models of TBI in rodents [12]. CCI allows control of relevant parameters related to the impact, such as contact velocity, dwelling time, and depth, to modulate the severity of damage [33]. Using this optimized model, we report that COs can recapitulate the primary pathology of TBI, including metabolic changes after neuronal damage, neuronal loss, and astrogliosis.

## 2. Materials and Methods

### 2.1. Derivation and Characterization of iPSCs from Human Fibroblasts

The work described in this study was approved by the institutional stem cell review committee at UThealth, Houston, TX. The generation of iPSCs from human dermal fibroblasts was carried out following the Cyto Tune-iPS 2.0 Sendai virus (SeV) reprogramming Kit (Thermo Fisher, A16517, Waltham, MA, USA). Briefly, MRC-5 human dermal fibroblasts, cultured to 90% confluency, were harvested after Accutase treatment for 4 min at 37 °C, and 150,000 cells were seeded in 0.1% gelatin-coated in one well in a 6-well plate and cultured overnight at 37 °C. At this stage, fibroblasts were transduced using the SeV cocktail in MEF medium (DMEM high glucose Sigma-Aldrich D5796, 10% FBS, Glutamax Gibco 25030081, MEM-NEAA Gibco 11140-050). Medium containing SeV was removed after 24 h, and the MEF medium was daily replaced for five days. Later, cells were re-plated into a 10 cm plate with MEF medium and cultured overnight. From day 6–13, cells were maintained with daily changes of the ReproTeSR medium (StemCell Technologies 05926, Vancouver, Canada). From day 14 and onward, cells were maintained with mTeSR1 medium (StemCell Technologies 85850). Reprogramed iPSC colonies were transferred separately to Matrigel-coated wells in a 12-well plate, maintained with mTeSR1, and kept growing in these conditions. Finally, iPSCs were grown on Matrigel-coated coverslips and phenotypically characterized for different pluripotency markers: alkaline phosphatase (AmsBio, StemAb Alkaline Phosphatase Staining Kit II 00-0055, Cambridge, MA, USA), following the manufacturer instructions, and immunofluorescence for the SRY-box transcription factor 2 (SOX2) (1:200, Abcam ab97959, Waltham, MA, USA), the Stage-specific embryonic antigen-4 (SSEA4) (1:200, Abcam ab16287), and the Octamer-binding transcription factor 4 (Oct4) (1:200, Stemgent 09-0023, Cambridge, MA, USA). Briefly, iPSCs were fixed with 4% paraformaldehyde in PBS for 15 min at 37 °C, washed with PBS, and incubated in blocking solution (3% BSA in 0.05% Triton X100 PBS) for 1 h at room temperature. Later, samples were incubated with antibodies diluted in blocking solution overnight at 4 °C. After washing with PBS, cells were incubated with fluorescent secondary antibodies; Anti-Mouse Alexa-594 (1:500, Invitrogen™ A32744, Waltham, MA, USA) or Anti-Rabbit Alexa-488 (1:500 Invitrogen™ A32790), stained with DAPI (4′, 6-diamidino-2-phenylindole), and covered with FluorSave (Millipore Cat 345789, Burlington, MA, USA) mounting medium.

### 2.2. Cerebral Organoid Generation

iPSC cells were maintained with mTeSR™ Plus (StemCell Technologies 05825) medium in plates coated with Matrigel Growth Factor Reduced Basement Membrane Matrix (Corning 354230, Corning, NY, USA) until complete stabilization. COs were initiated when iPSC colonies reached homogenous groups with <10% of differentiated cells, utilizing the STEMdiff™ Cerebral Organoid Kit (StemCell Technologies 08570), as recommended by the manufacturer, with little modification. Briefly, at day zero, iPSC cells (confluency ≤ 80%) were washed with PBS and then gently dissociated by adding TrypLE™ Express (GIBCO 2604021); iPSCs were resuspended in embryoid bodies (EBs) Formation Medium with Y-2763 at 10 µM. Cells were counted in a Neubauer hemocytometer and then placed at 9000 cells/well in a Corning 96-well round-bottom ultra-low attachment microplate (Corning CLS7007). The plate was placed at 37 °C without disturbing it for 24 h. On days two and four, the resulting EBs were fed with Formation Medium without Y-2763. On day five, EBs were individually and carefully transferred into each well of a Costar 24-well ultra-low attachment plate (Corning CLS3473) containing Induction Medium. On day seven, each EB was embedded in 15 µL of Matrigel hESC-Qualified Matrix (Corning 354277) and placed into the incubator at 37 °C for 30 min. After incubation, 12 to 15 Matrigel-embedded EBs were placed in a 6-Well Ultra-Low Adherent Plate (Corning CLS3471) containing Expansion Medium. By day 10, Expansion Medium was replaced with Maturation Medium. Finally, changes in the maturation medium were performed every three days, until the CCI procedure was done at 220 days in vitro (DIV).

### 2.3. Animal Experiments

Two months old wild-type mice (C57Bl6/J) were utilized to evaluate the effect of CCI and used as a positive control. All animal procedures described in this article were approved by the Center of Laboratory Animal Medicine and Care (CLAMC) and the Animal Welfare Committee (AWC) of the McGovern Medical School, University of Texas Health Science Center at Houston.

### 2.4. Controlled Cortical Impact Procedure in Live Mice

Mice were deeply anesthetized with 5% isoflurane in an induction chamber and transferred to a stereotaxic frame and maintained under 2% isoflurane. Ophthalmic ointment was applied to both eyes. The mouse head was clipped free of hair, and the skin was surgically prepped, performing three alternated scrubs of iodine and 70% isopropanol. A subcutaneous injection of bupivacaine was administrated along with the incision site. An incision of ~1–1.5 cm was made, and the skull was exposed. Approximately 4 mm diameter craniotomy was performed using a 10,000 RPM drill and a 2 mm drill bit to expose the brain cortex for the CCI procedure. The impact was carried out using an Impact One Stereotaxic Impactor (Leica Biosystems, Buffalo Grove, IL, USA) attached to the stereotaxic frame. Impact parameters were calibrated based on previously reported protocols [34,35] considering velocity (4 m/s), dwell time (200 ms), and depth (1 mm). After the impact, the skin was closed using monofilament sterile suture (6-0-non-absorbable), and 0.9% sodium chloride was given intraperitoneally (IP). Animals were transferred to a recovery cage where additional heat support was provided until fully mobile. Seven days after the CCI procedure, mice were euthanized by CO_2_ inhalation and perfused with cold 1× PBS, containing 5 mM EDTA, and brains were collected for analysis.

### 2.5. Phantom Brain Development

Currently, COs can not generate a structure comparable to the size of mice brains. Therefore, to perform CCI in COs under standard parameters, an adequate cushion-like substrate was required. To this extent, we first analyzed the mechanical properties of the mouse brain to create an adequate substrate for our model. Mouse brains were analyzed in two different dynamic scenarios. First, brains were subjected to uniaxial compression assays using a slow compressive load rate (180 µm/s). At the moment of the compression, brains were placed on top of a calibrated sensor or load cell. Once compression started, the load transmitted through the brain to the sensor was measured in grams and plotted in real-time. This assay allowed us to measure the ability of the brain to transmit the applied compressive load, thus working as an estimation of brain stiffness. Secondly, we evaluated the response of brains under CCI conditions, using a fast impact (4 m/s) with a depth of 1 mm. Similarly, the peak of the transmitted load at impact was measured in grams, which we refer to as impact transmission. With these two measurements, we established basic baselines for further development of a phantom brain, using a modification of previously published agarose-based brain-like mixtures [36,37]. Mixtures were prepared using agarose LE (Thomas Scientific™, Swedesboro NJ, USA) and gelatin from porcine skin (Sigma-Aldrich™ G1890-500G, San Louis, MO, USA), weighed, diluted in sterile PBS, and boiled in a hot plate. Once melted, the mixtures were vortexed and placed in molds, with a volume comparable to a whole mouse brain. The mixtures were analyzed with the same two approaches previously described above to find the best match between the mouse brain and the agarose-gelatin mixtures.

### 2.6. Mouse Skull Preparation for CCI

A real bone-skull derived from a previously euthanized mouse was carefully anatomically prepared as a reservoir for the phantom brain (Supplementary Figure S1). The skull was processed with modifications of a previously described protocol [38] based on hydrogen peroxide bone cleaning and clearing procedures. Briefly, after collecting the mouse head, large soft tissue was removed using surgical tools. Subsequently, the sample was incubated overnight with 30% hydrogen peroxide, followed by three consecutive washes in PBS. Afterward, tissue remains were carefully removed. To avoid leakage of the liquid state of the phantom brain, specific areas on the skull were sealed with dental cement; palatine process, Cranio-pharyngeal channel, tympanic bulla, and the foramen Magnus. Meanwhile, the external auditory meatuses were left uncovered to fit the ear bars from the stereotaxic frame. To complete the skull preparation, two circular windows of ~4 mm in diameter were drilled bilaterally, one in each parietal bone.

### 2.7. Controlled Cortical Impact Procedure in COs

A stereotaxic frame was disassembled and sterilized using hydrogen peroxide steamed gas. Once the sterilization procedure was completed, the frame was re-assembled in a biosafety cabinet. The sterile mice skull was filled with the Phantom brain or Mix 3 and kept in the biosafety cabinet to solidify for ~15 min. Once solidified, the skull was mounted in the stereotaxic frame and secured with ear and tooth bars. COs were carefully transferred using a sterile stainless spoon on top of the phantom brain through the skull windows previously drilled (Supplementary Figure S1). The CCI equipment was calibrated to deliver a mild to severe impact, following previously described parameters. A built-in contact sensor was used to establish the zero on the z-axis. Once the procedure was completed, impacted and controls COs (Total *n* = 6) were carefully transferred into a 6-Well ultra-low adherent plate, washed with PBS, and fed with fresh maturation media for 7 days at 37 °C. The media tested negative for bacterial contamination.

### 2.8. Immunofluorescence

Injured mouse brains and controls were collected 7 days post-impact, fixed in 4% formaldehyde solution for 72 h. Similarly, COs were removed at 7 days post-impact from maturation media, fixed in 4% formaldehyde solution for 24 h. Both COs and brains were serially dehydrated with EtOH, paraffin-embedded, and sliced in 10 μm-thick serial sections and processed for immunostaining. Sections were deparaffinized/rehydrated and treated with 3% BSA in 0.2% Triton X-100 PBS to block nonspecific antibody binding. Sections were incubated overnight with Anti-SRY-box transcription factor 2 (SOX2) (1:200, Abcam™ ab97959), Anti-Tubulin β3 (1:400 BioLegend 801201), anti-Forkhead box protein G1 (FOXG1) (1:400, Abcam™, ab18259), Anti--box brain transcription factor 1 (Tbr1) (1:200, Millipore™, ab100554), Anti-Special AT-Rich Sequence-Binding Protein 2 (SATB2) (1:200, Abcam™, ab34735) Anti-GFAP (1:500, Abcam™ ab7260), Anti-MAP2 (1:400, BD Pharmingen™ 556320), Anti-NSE (1:1000 Proteintech™ 10149-1-AP), or Anti-Cleaved Caspase 3 (1:500 Abcam™ ab2302) primary antibodies. Respective secondary Anti-Mouse Alexa-594 (1:500, Invitrogen™ A32744) or Anti Rabbit Alexa-488 (1:500 Invitrogen™ A32790) antibodies were incubated for one hour. Sections were examined by fluorescence microscopy (DMI6000B, Leica Microsystems, Buffalo Grove, IL, USA), photomicrographs were taken with a digital camera (360FX Leica) and imported into ImageJ 1.45 s software (NIH) for analysis. Mouse brain photomicrographs were analyzed across the penumbra surrounding the impacted zone. Similarly, zonification of the Cos was performed to determine the region of interest. Divided as the cortical zone (Z1), a transition zone (Z2A), and a necrotic core (Z2B) characterized by the absence of MAP2 positive cells at the inner region of the COs and a dense DAPI staining (S.4).

### 2.9. Statistical Analysis

Graphs were expressed as means ± standard error of the mean (S.E.M). Student’s *t*-test was used to compare GFAP integrated density normalized by immunoreactivity area, MAP2 integrated density of binary mask area normalized by DAPI staining. Corrected total cell fluorescence of NSE (CTCF = Integrated Density—(Area of selected cell X Mean fluorescence of background readings), Cleaved Caspase 3 ratio of total cells, and Transmitted force during CCI impact. Nonlinear curve fit analysis was applied to evaluate load transmission during uniaxial compression studies. Statistical differences were considered significant at the *p* ≤ 0.05 level. Statistical analysis was performed using Graph Pad Prism 5.0 software (GraphPad Software Inc. San Diego, CA, USA).

## 3. Results

### 3.1. Generation and Characterization of the Phantom Brain

**Figure 1:**
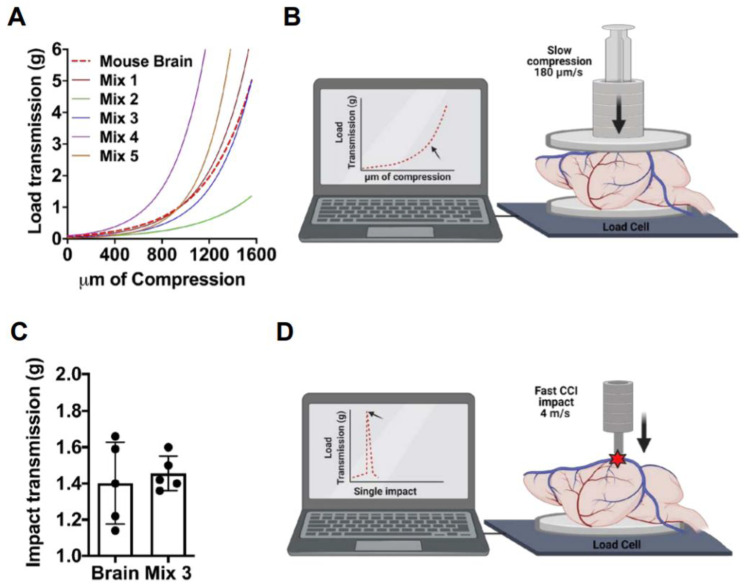
Phantom brain development. Phantom brain and mouse brains were analyzed and compared using slow uniaxial compression assay and fast impact assay. (**A**,**B**). Visualization of the non-linear curve fit models generated from the different preparations and mouse brains analyzed by a slow (180 μm/s) uniaxial compression assay to evaluate stiffness. Non-linear fit test of Phantom brain Mix 3 resulted in a shared curve model equation Y = 0.06650 * exp(0.002669 * X), r^2^ 0.9680; *p* = 0.9651; n = 3. (**C**,**D**). Impact transmission of CCI at 4 m/s, performed in the mouse brain, and compared to the phantom brain (Mix 3) n = 5. Phantom brain (1.456 g ± 0.09) and mouse brain (1.402 g ± 0.22) displayed a similar response to CCI (Student *t*-test; *p* = 0.6453).

The standard platforms to perform CCI in living animals require a stereotactic frame to secure the mouse’s skull in a fixed position [12]. To translate the standardized mechanical parameters to our COs, we decided to use a mouse skull to support the CCI model in COs, coupled with the design of a phantom brain physically similar to the mouse brain to be used as beading for COs. We designed our phantom brain mixture based on previous reports showing that agarose polymers at certain concentrations can mimic the stiffness of a mammalian brain [36]. To identify the best material to mimic the brain, different agarose/gelatin-based mixtures were prepared (Table 1). We have evaluated the mechanical responses of the brain and the different mixtures with two dynamic scenarios. First, we performed a slow uniaxial compression assay (180 um/s). This procedure allowed us to measure and compare the stiffness of the brain with the five different agarose-based mixtures (Figure 1A,B). With these data, we performed a nonlinear curve-fit test of each compression response compared with the brain curve. As a result, Mix 3 (0.8% gelatin and 0.3% agarose), hereafter called the phantom brain, was able to best fit the curve of the mouse brain (r^2^ 0.9680; *p* = 0.9651; *n* = 3). Secondly, we proceeded to evaluate and compare the mechanical response of the brain and phantom brain to a fast compressive load (4 m/s) and the same parameters of the CCI impact previously described. We measured the peak of the transmitted load in grams through the analyzed samples. This assay demostrated that the response of the brain and phantom brain to the impact parameters of CCI did not showed significant differences (Student *t*-test; *p* = 0.6453) (Figure 1C,D). Altogether, both assays, first a slow compression assay and second a fast impact, validated our Mix 3 as the phantom brain required to adapt the CCI model to COs.

### 3.2. Generation and Characterization of Human iPSCs and COs

**Figure 2:**
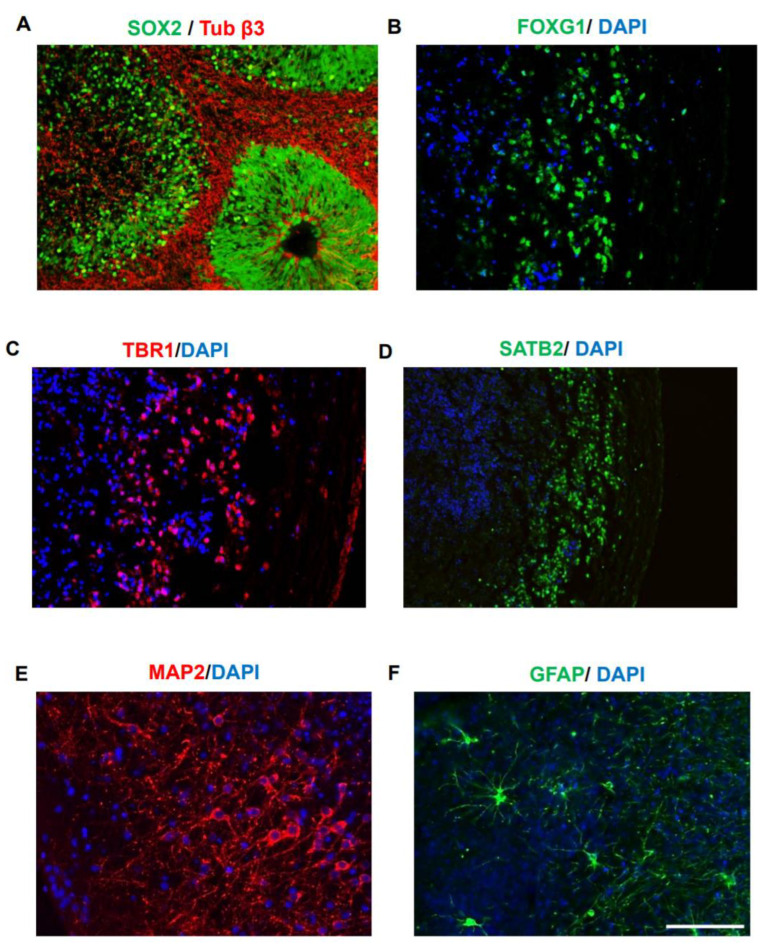
Generation of cortex-like cerebral organoids. COs were generated from a healthy iPSC line as previously described and characterized at 44 DIV and 220 DIV. (**A**). Characterization performed at 44 DIV indicated Sox2 positive ventricular zone (VZ) formation and β3 tubulin (Tuj1) positive neurons (in red) in the basal surface. Neuroepitelium-like structures similar to those seen in the brain during early stages of development were observed. (**B**–**F**). Characterization of COs at 220 DIV. FOXG1 immunostaining was used to confirm forebrain density (**B**). Appearance of cortical layer formation is analyzed using TBR1 (layer IV marker) (**C**) and SATB2 (layer II/IV specific marker) (**D**). The appearance of fully differentiated neurons and astrocytes was analyzed by immunostaining with MAP2 (**E**) and GFAP (**F**), respectively. The scale bar is 100 µm (showed in panel (**F**)) for all the images except （**D**）. For （**D**）, the scale bar is 200 µm.

Human fibroblasts were reprogramed using Cyto Tune-iPS 2.0 Sendai virus (SeV) reprogramming kit. iPSC colonies showed the expected morphology (Supplementary Figure S2A) and were characterized using alkaline phosphatase activity (Supplementary Figure S2B). The expression of pluripotency markers SOX2, SSEA4, and OCT4 was observed (Supplementary Figure S2C). COs were generated using STEMdiff protocol following the instructions from Stem Cell Technologies. Uniform embryoid bodies were generated from aggregated iPSCs with a sharp edge and translucence neuroectoderm, which upon neural induction and matrigel embedding, produced multiple neuroepithelial buds. Morphometric analysis at 44 DIV indicated that COs generated a readily oriented SOX2 positive ventricular zone surrounded by early neurons (Figure 2A). Later, at ~220 DIV, forebrain identity was confirmed by immunostaining with FOXG1 (Figure 2B). At this time, COs displayed signs of cortical layer formation, evident by immunostaining with layer VI- and IV-specific marker TBR1 (Figure 2C) and SATB2 (Figure 2D), as previously published [22]. At this stage, COs also displayed MAP2 positive neurons (Figure 2E) and GFAP positive astrocytes resembling mature morphology (Figure 2F). To investigate the variability of different preparations of COs and based on the observed radial symmetry, we estimated a coefficient of variability for the radial extent of MAP2 and GFAP immunoreactivity in five independents organoids (Table 2), showing that there was no significant variability among distinct organoids in terms of the populations and distribution of neurons and astrocytes.

### 3.3. CCI Induces Astrogliosis and Reduces Neurons in COs

**Figure 3:**
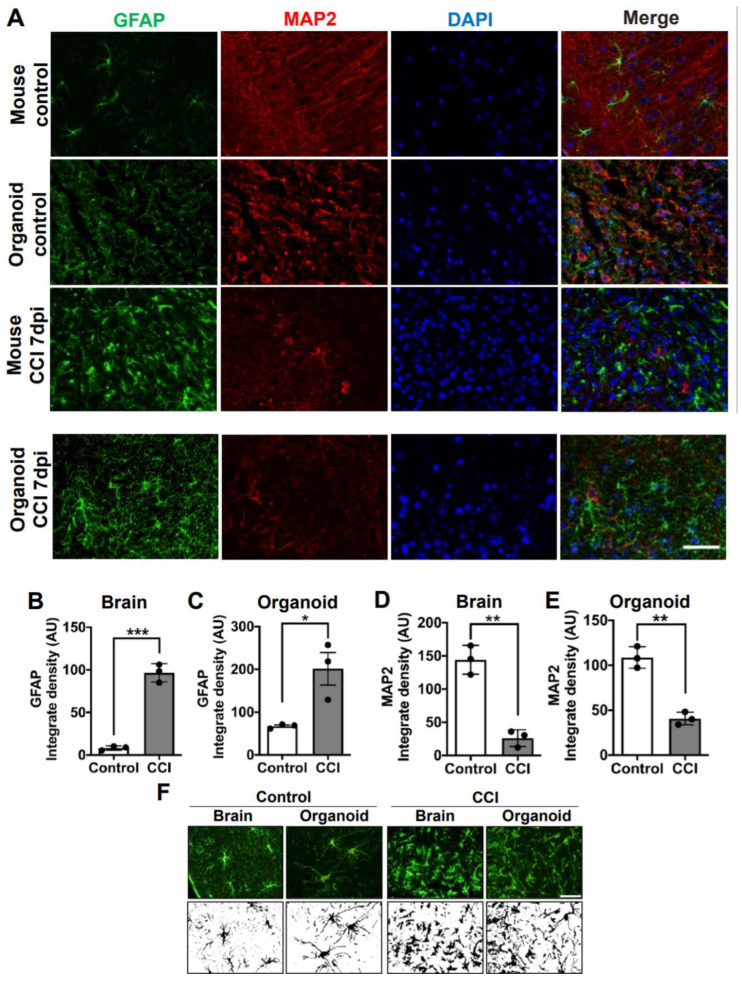
Astrogliosis and reduction of neurons in COs after CCI. (**A**) Microphotographs of COs and mice brain subjected to CCI stained with GFAP and MAP2 antibodies to recognize astrocytes and neurons, respectively. Immunostaining was done 7 days after CCI. (**B**) Immunofluorescence quantifications of GFAP in mouse brain (Controls 8.241 ± 2.5 vs. CCI 96.68 ± 10.7; *p* = 0.0002) and (**C**) COs (Controls 67.31 ± 5.0 vs. CCI 201.6 ± 65; *p* = 0.0241). MAP2-positive neuronal density in (**D**) mouse brain (Control 144.2 ± 21.7 vs. CCI 26.24 ± 12.5; *p* = 0.0012) and in COs (**E**) (Control 108.7 ± 11.9 vs. CCI 40.73 ± 7.0; *p* = 0.001). (**F**) Morphological changes in astrocytes of COs and mouse brains were observed 7 days after CCI. Magnification: X40, scale bars = 50 μm. Statistical analysis performed with Student’s *t*-test, * *p* ≤ 0.05; ** *p* ≤ 0.01; *** *p* ≤ 0.001.

To model TBI in COs, we delivered the impact into COs embedded in the mouse skull and supported by the phantom brain. CCI was performed in COs at 220 DIV using our newly adapted method. As sham controls, we placed the COs in the skull filled with the phantom brain without the impact. The CCI method is well-established to model moderate to severe TBI in mouse. Thus, as a positive control, we also applied CCI into a live mouse brain to compare with COs. To assess astrogliosis, we performed immunofluorescence analysis using glial fibrillary acid protein (GFAP) as an astrocyte marker to evaluate changes in expression and morphology. In the control mouse brain, astrocytes displayed a healthy ramified morphology (Figure 3A,F). Upon CCI, astrogliosis in mouse brains was apparent and characterized by a hypertrophic morphology in the astrocytes, with an increase in GFAP immunoreactivity area (Figure 3A,F). Image quantification indicated that there is a significant increase in GFAP immunoreactivity in the mouse brain after CCI compared to sham control (Figure 3B). Similarly, astrocytes in sham-operated COs also displayed longer branched processes matching a classic stellate morphology [39] indicative of a resting state (Figure 3A,F). As expected, human astrocytes were significantly larger than mouse astrocytes (Figure 3F), corroborating their hominid nature [40]. Remarkably, CCI also induced a significant increase in GFAP immunoreactivity in COs compared to sham-operated controls (Figure 3A,F and Supplementary Figure S3). Furthermore, GFAP positive cells in CCI-impacted COs displayed hypertrophic process combined with the loss of branching and broadening of process reminiscent of activated astrocytes (Figure 3A,F and Supplementary Figure S3). Image quantification confirmed that there is indeed a significant increase in GFAP immunoreactivity in CCI-impacted COs compared to sham controls (Figure 3C). These data indicated that our CCI-based model in COs can recapitulate astrogliosis, one of the key features of TBI. We also noted a significant decrease in MAP2 immunoreactivity in mice exposed to CCI compared to sham controls (Figure 3A,D). Interestingly, COs exposed to CCI displayed a similar significant reduction in MAP2 positivity in comparison to sham controls, indicating a possible loss of neurons (Figure 3A,E and Supplementary Figure S3). Excitingly, the magnitude of astrogliosis and reduction in postmitotic neuronal marker after CCI was comparable between the COs and mouse model, supporting our newly adapted methodology to study TBI in vitro.

### 3.4. Elevated Neuronal Damage in COs after CCI

**Figure 4:**
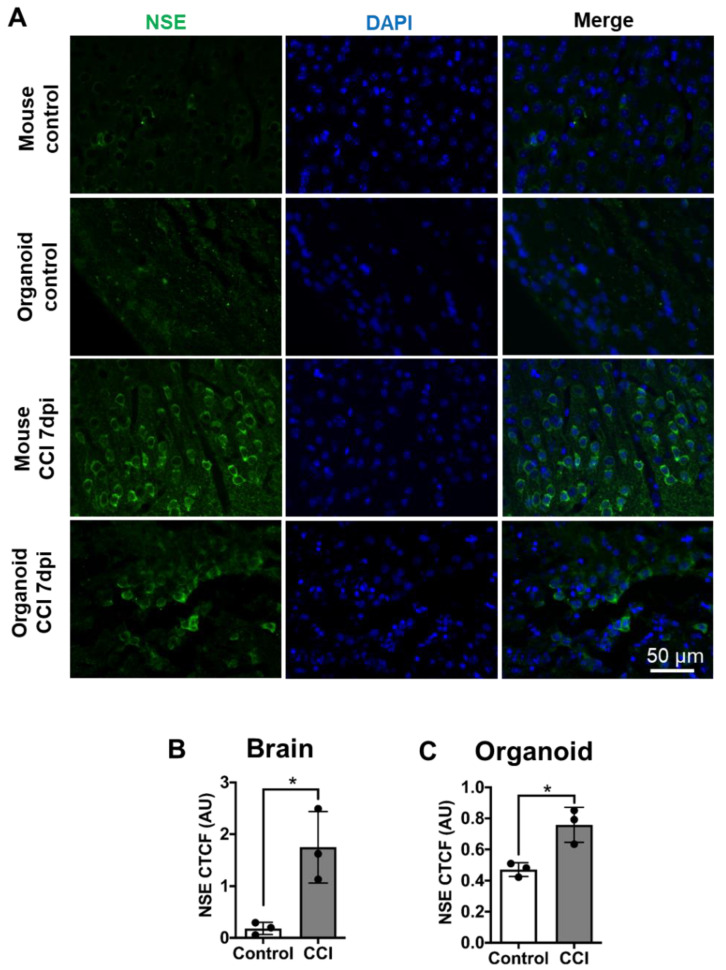
CCI induced neuronal damage in COs. (**A**) To analyze neuronal damage after CCI, mouse brain and COs slides were immunostained with NSE antibody. Images were analyzed using corrected total cellular fluorescence. (**B**) Significant increase in NSE staining in mouse brain after CCI compared to control. (**C**) Similarly, CCI in COs significantly increased NSE accumulation in cells compared to sham control, indicating neuronal damage. Statistical analysis performed with Student’s *t*-test, * *p* ≤ 0.05.

Neuronal damage is one of the hallmark primary pathological features of TBI. We analyzed neuronal damage in COs, 7 days after CCI using neuron-specific enolase (NSE). NSE, an enzyme involved in glycolysis, has been reported as a marker of late neural maturation [41] and is considered a biomarker that can directly assess functional damage to neurons [42,43]. Moreover, NSE expression levels have a positive correlation with the severity of TBI [44,45]. The basal level of NSE immunostaining in the control mouse brain and sham-treated COs were comparable (Figure 4A). As expected, CCI induced neuronal damage in the mouse brain, which was evident by cytosolic accumulation of NSE (Figure 4A). Indeed, image analysis indicated NSE accumulation significantly increased in mouse brains after CCI compared to controls (Figure 4B). Excitingly, we noted a similar pattern of NSE accumulation in the COs impacted by CCI indicating neuronal damage (Figure 4A and Supplementary Figure S4). Furthermore, image quantification indicated that CCI-impacted COs accumulated significantly higher levels of NSE compared to sham-operated COs (Figure 4C), although the increase was smaller as in mouse brain after CCI. These data indicate that our model of CCI is effective to induce neuronal damage in COs.

### 3.5. Cellular Apoptosis in COs after CCI

**Figure 5:**
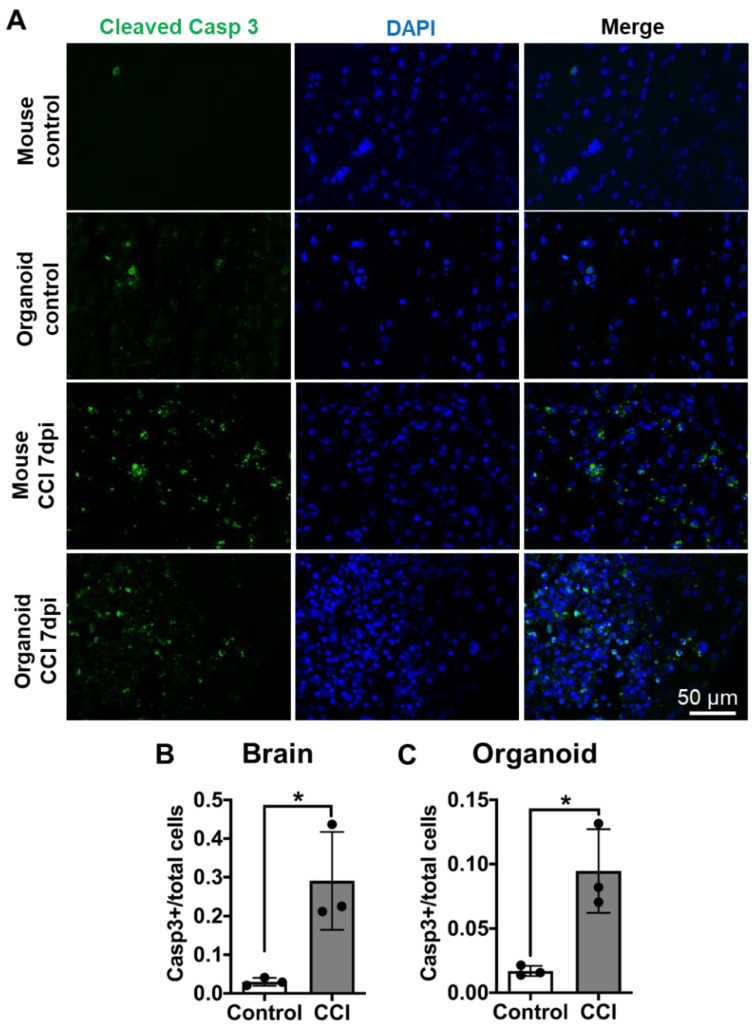
CCI-induced apoptosis in COs. (**A**) To analyze the induction of apoptosis, we immunostained sections from mouse brain and COs with an antibody raised against cleaved caspase 3, a well-established marker of apoptosis. Mouse brains were analyzed across the impact zone and penumbra. (**B**) Significant increase in cleaved caspase 3 in mouse brain after CCI compared to control. (**C**) Similarly, CCI significantly increased NSE accumulation in COs compared to sham control, indicating neuronal damage. Statistical analysis performed with Student’s *t*-test, * *p* ≤ 0.05.

Depending on the severity of the damage, neuronal loss by apoptosis can be evident in the primary phase of TBI [46]. To further confirm that there is cell death after CCI, we analyzed caspase activation using cleaved caspase 3 as an apoptosis marker [7,47]. We observed in both COs and mouse brain that CCI treatment increased the extent of cleaved caspase 3 immunostaining (Figure 5A). Image analysis showed that the magnitude of apoptosis increased by CCI was very comparable between mouse brain and COs (Figure 5B,C).

## 4. Discussion

Several in vitro and in vivo models have been developed to study the cellular and molecular events associated with TBI. In vitro models include stretch- and shear-based in vitro culture systems employing different types of cells, including neurons derived from human iPSCs [15,16,17]. However, these 2D-based culture models do not have the 3D cellular organization and complexity of the brain nor the extracellular matrix composition of the brain. These are important weaknesses since TBIs likely involve structural damage to the brain and affect various different cell types and networks of cellular interaction. To overcome the problems with cellular models, several methods have been developed for experimental induction of TBI in animal models. The most widely used models include subjecting mice to fluid percussion injury, weight drop–impact acceleration injury, blast injury, and CCI [14]. The problem with these models is that the human and mouse brains are anatomically, morphologically, structurally, and functionally very different [48]. This led us to attempt to develop a new in vitro model of TBI using human cells organized in a 3D architecture similar to the human cortex. In this study, we developed a human brain organoid-based model of TBI. To this end, we have adapted the CCI method, originally developed for small rodents [12,14,34,47], to COs, using a phantom mouse brain and a mouse skull. We chose the CCI model since this technique is well-established and allows independent control of contact velocity and level of deformation of the brain [33]. We were able to calibrate the equipment to perform a mild to severe TBI in COs placed on top of phantom brain preparation. Agarose/gelatin-based gels have been used in different disciplines of neurology research [49] and are widely accepted as viable in vitro models of the physical characteristics of the human brain [36]. In this work, we identified the best agarose-based polymer to reproduce the stiffness and density of the mouse brain. Although our present studies were done using an open skull procedure, further developments to apply the impact without the need for a skull window (closed skull injury) are ongoing.

TBI pathology displays a complex spatiotemporal gradient of events involving multiple brain cell types [50,51,52]. To this extent, human COs displays the remarkable heterogeneity of human brain cells. Even more, different cell types maintain their spatial organization, providing a great opportunity to model and understand the complex pathological cascade of TBI [23]. In this work, we demonstrate for the first time, that the optimized CCI method can induce hallmark features of TBI in COs, including neuronal damage, neuronal loss, and astrogliosis.

Human COs provide the opportunity to model pathology within a human genome. This is a point of great interest, especially regarding TBI pathology, which involves a variety of genetic networks. Different genetic polymorphisms can be associated with differential prognostic outcomes in TBI [53,54]. For example, *APOE* gene polymorphism is significantly associated with the development of Alzheimer’s disease (AD)-like dementia after TBI [55]. The fact that COs can be generated from iPSCs derived from human donors carrying specific polymorphisms or mutations suggests that the TBI-organoid model can be used to dissect the role of particular gene variants in disease pathology and even predict the pathological outcome of TBI using a personalized medicine approach. Furthermore, the flexibility to generate a large number of organoids in vitro may provide a unique platform for drug screening to prevent TBI-induced brain damage.

CCI-impacted COs displayed a damage response in different nerve cells, a key feature of the primary response to TBI. Remarkably, the involvement of cell types and the response at the analyzed time point after injury were comparable between the in vitro generated human COs and the in vivo mouse model, which supports the idea that COs are biologically relevant for TBI research. Metabolic changes are reported to occur in neurons after TBI. Brain injury affects neuronal circuitry by causing damage and death of neurons, destroying connections between them, affecting dendrites and axons [52]. This can lead to excessive accumulation of neurotransmitters in the brain tissue, in particular glutamate, which can overstimulate neurons and cause further damage [7,52]. TBI results in an immediate increase in glucose cerebral metabolic rates [56]. To investigate this, we evaluated the levels of NSE, an enzyme involved in glycolysis, reported as well as a marker of late neural maturation [41] and considered as a biomarker that can directly assess functional damage to neurons [42,43]. Moreover, NSE expression levels have a positive correlation with the severity of TBI [44,45]. Our results indicate that the CCI procedure applied in this protocol causes a significant steady-state accumulation of NSE and reduced MAP2 immunoreactivity for postmitotic neurons in COs. In fact, the magnitude of predicted neuronal loss was strikingly similar between COs and mice brain impacted by CCI. Corroborating these findings, we also found a significant increase in apoptotic cells in COs after CCI comparable to the changes observed in the mice model. Altogether, our results suggest that COs at 220 days old harbor mature neurons that can recapitulate various cerebral abnormalities associated with TBI. Further studies of metabolic changes produced by TBI at later time points, including accumulation of misfolded protein aggregates, perturbation of cellular calcium homeostasis, increased free radical generation, lipid peroxidation, and mitochondrial dysfunction [57], are required to explore the use of COs as a model of the secondary injury associated with TBI.

Among all the cell types in the brain, astrocytes are the most ubiquitous throughout brain tissue and make essential contributions to several homeostatic functions that could directly influence neuronal survival and tissue integrity [58]. Astrocytes are one of the key responders to damage evoked by TBI and play a crucial role in determining the functional outcome of the damage [5,59]. These cells are phenotypically characterized by a stellate morphology, which changes to a reactive hypertrophic state under stress [39,60] and degenerative conditions [61]. To evaluate the reactivity of astrocytes in COs after CCI, we analyzed the expression changes of GFAP [59]. The changes in the expression of GFAP in COs 7 days after the CCI procedure correlate with the reactive state of astrocytes. These results provide evidence that supports the functional and biological relevance of astrocytes generated in COs for TBI research. However, further studies need to be performed to describe the pathways involved and their translational applicability.

One of the main limitations of COs is that they do not have all the brain cell types (e.g., they lack microglial cells) at the proportions found in the human brain. They also lack vasculature. Consequently, we were unable to model some of the crucial features of TBI, such as microglial activation, cerebral hemorrhages, and edema. Nonetheless, COs technology is a fast-growing field, and several research groups are developing protocols to enrich brain organoids with different cell types, such as microglia and oligodendrocytes [62,63,64]. Future developments should also enable generating and fusing different brain regions to model neuroanatomical connections [63,65] and producing organoids with vasculatures [66,67]. It is also conceivable that human COs could be implanted into living mice. TBI protocols applied on successfully implanted COs in live mouse brains, may allow studying in vivo the response to TBI in human cells. These advances may provide a unique opportunity to dissect the brain cell type region and vasculature role in TBI pathology and its transition from primary to secondary damage. Our work developing a novel platform for TBI, reproducing some of the key primary pathological features of TBI in a human cortex-like brain structure, offers a promising opportunity to study not only the cellular and molecular changes responsible for brain damage after TBI but also to evaluate different therapeutical approaches to treat adult and pediatric TBI in collaboration with specialized clinical centers of TBI research.

## Figures and Tables

**Table 1:** Phantom brain preparations.

	Mix 1	Mix 2	Mix 3	Mix 4	Mix 5
Gelatin %	0.6	0.6	0.8	1.5	1
Agarose %	0.4	0.1	0.3	0.5	0.8

**Table 2:** Calculations of coefficient of variation for the population of neurons and astrocytes in COs, as measured by MAP2 and GFAP staining. Data are shown as radial coverage (µm) in COs.

	**Neurons**			**For Each Organoid**			**All Together**		
				**Mean**	**SD**	**CV**	**Mean**	**SD**	**CV**
Org 1	315	324	343	327.33	14.295	4.367	333.87	13.98	4.18
Org 2	337	319	346	334	13.748	4.1161			
Org 3	318	301	325	314.67	12.342	3.9224			
Org 4	347	356	323	342	17.059	4.9879			
Org 5	339	367	348	351.33	14.295	4.0686			
	**Astrocytes**			**For Each Organoid**			**All Together**		
				**Mean**	**SD**	**CV**	**Mean**	**SD**	**CV**
Org 1	441	443	476	453.33	19.655	4.3357	507	52.4	10.34
Org 2	606	598	576	593.33	15.535	2.6182			
Org 3	468	495	503	488.67	18.339	3.7529			
Org 4	478	504	485	489	13.454	2.7513			
Org 5	502	512	518	510.67	8.0829	1.5828			

## Supplementary Materials

The following are available online at https://www.mdpi.com/article/10.3390/cells10102683/s1, Figure S1: Graphic representation of the CCI adaptation procedure for COs, Figure S2: iPSC generation and characterization, Figure S3: Low, Mid and High-power magnification of COs immunostained for MAP2 and GFAP, Figure S4: MAP2 and NSE colocalization, Figure S5: Zonification for analysis of Cos.
