# Supplementary material for: Modeling Traumatic Brain Injury in Human Cerebral Organoids
Source: Cells. 2021 Oct 7;10(10):2683. doi: 10.3390/cells10102683 (PMC8534257; doi:10.3390/cells10102683)
Supplement: Supplementary file 1 [file cells-10-02683-s001.zip › cells-1363877-supplementary.pdf]

Supplementary Figures

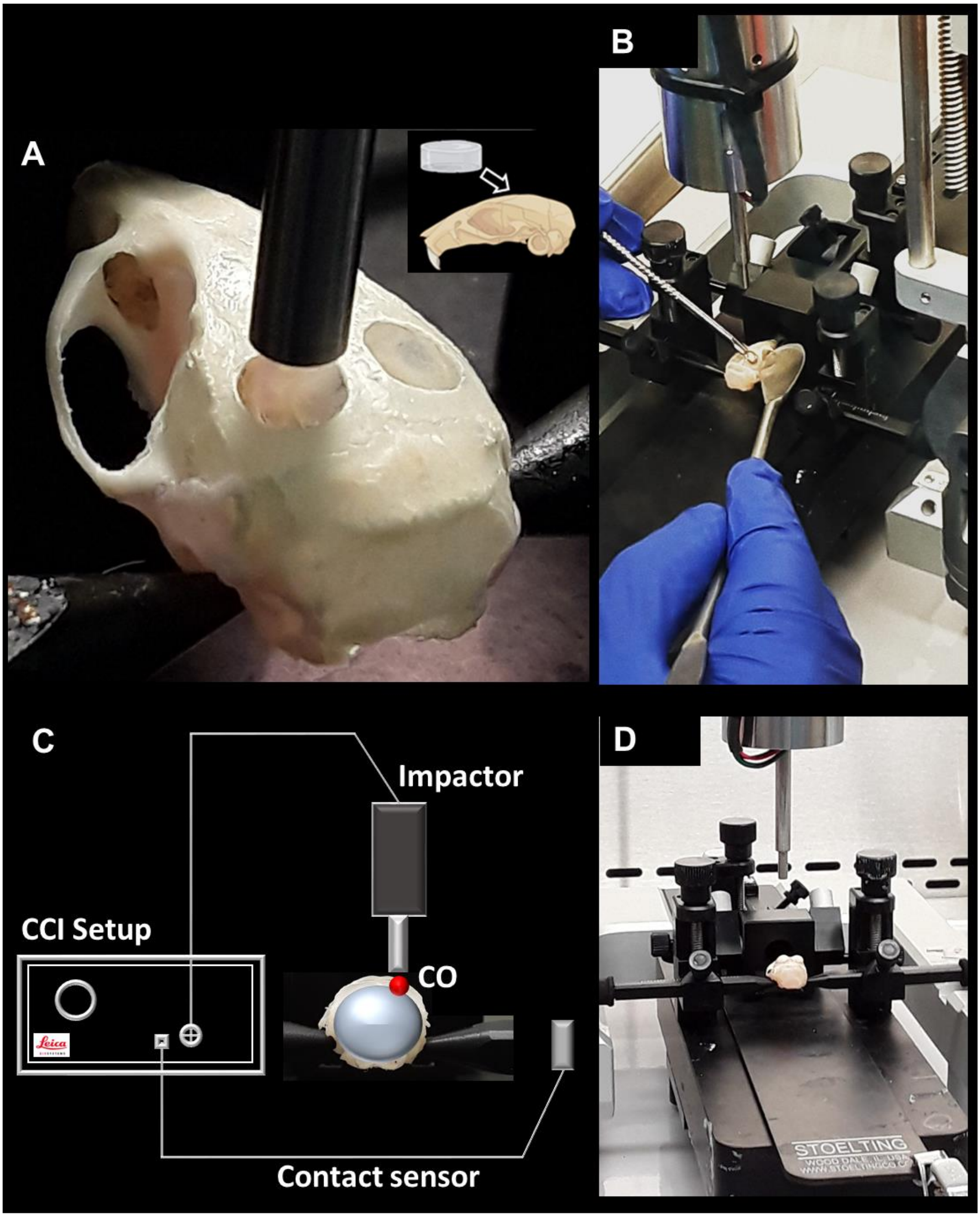

Figure S1. Graphic representation of the CCI adaptation procedure for COs. **(A)** Mouse skull and stereotaxic frame showing ~ 4mm bilateral cranial windows. **(B)** Skull was sealed with dental cement and loaded with the Phantom brain (Mix 3). **(C)** COs location in the solidified phantom brain through the skull window. **(D)** CCI Impact under stereotaxic apparatus.

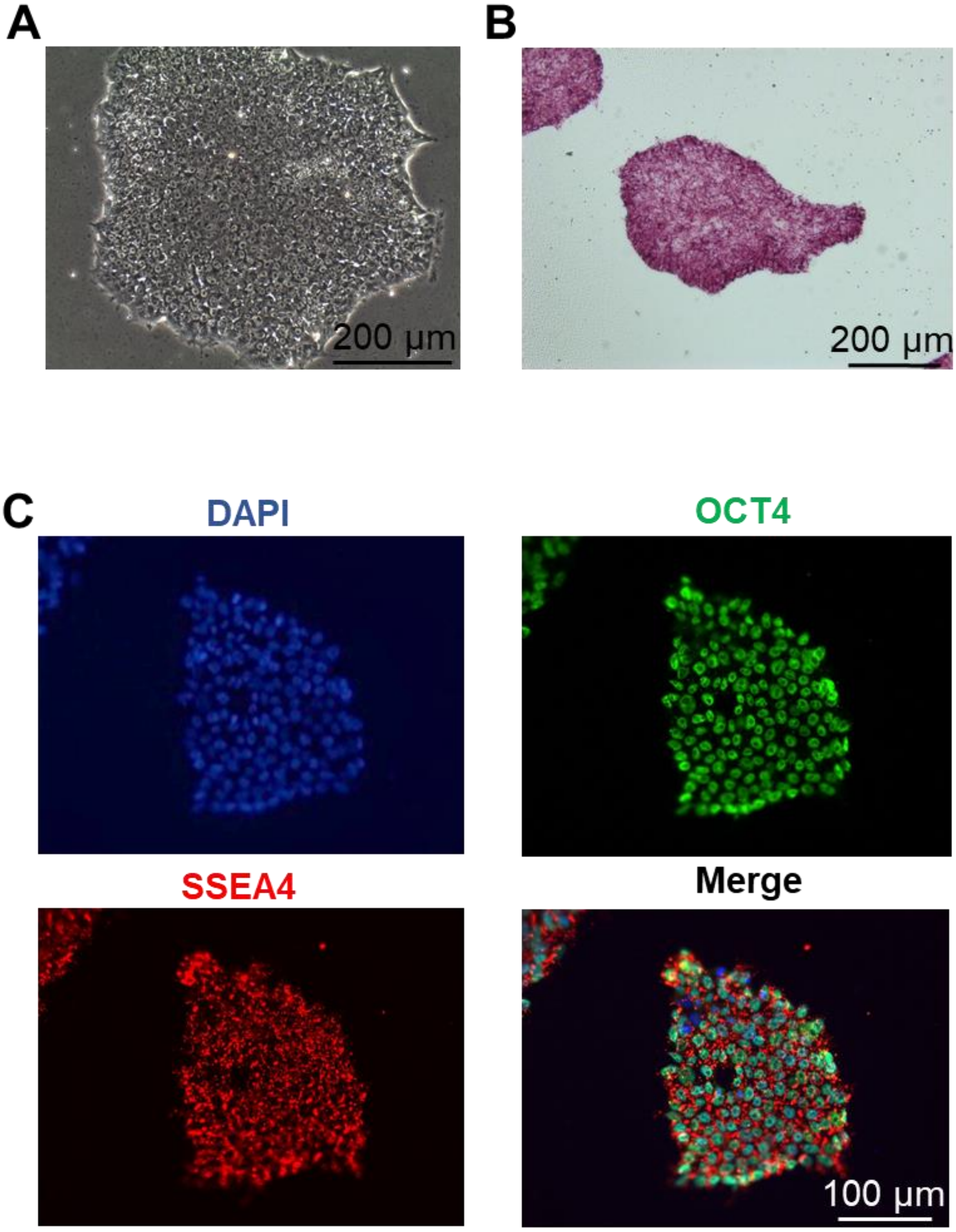

Figure S2. iPSC generation and characterization**.** Human skin fibroblasts were reprogrammed using CytoTune-iPS 2.0 Sendai reprogramming kit. A fully reprogrammed colony demonstrated compact architecture with sharp boundaries **(A)** and high levels of alkaline phosphatase (AP) **(B)**. The reprogramed colonies also expressed pluripotency markers including Oct4 (green) and SSEA4 (red) **(C)**.

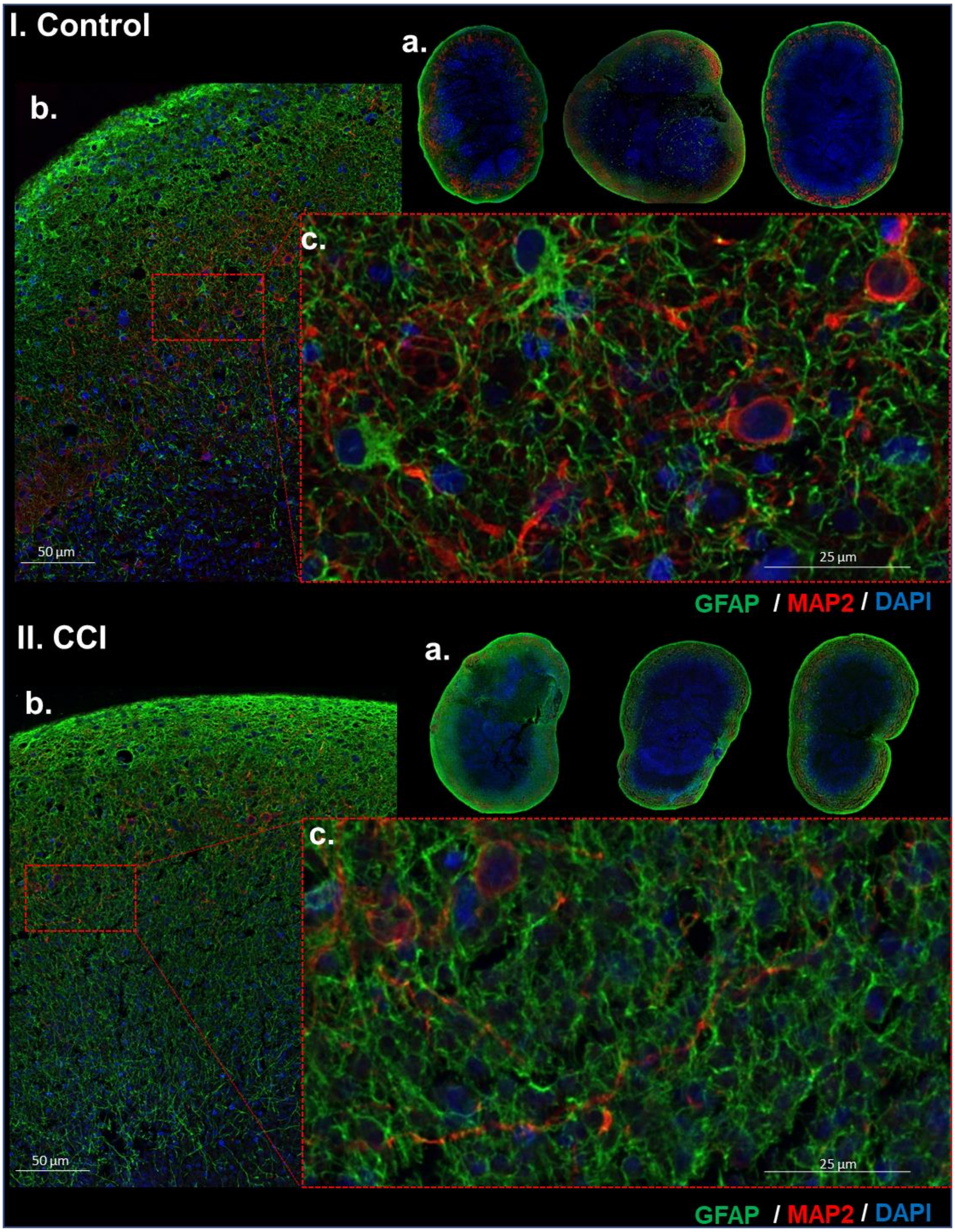

Figure S3. Low, Mid and High-power magnification of COs immunostained for MAP2 and GFAP. Low power magnification of control organoids (**I. a**) showing conserved morphology and radial distribution of MAP2 or GFAP positive cells across the cortex, (CV 4.18% and CV 10.34% respectively, calculated with an n = 5; Table 2). MAP2 and GFAP determined the region of interest (ROI). The necrotic core at the inner zone of the COs characterized by the absence of MAP2 and GFAP positive cells was excluded from the analysis. Evidently, GFAP was increased in CO after CCI (**II. a**). Mid power magnification images showed the cellular distribution across the cortex layer of control organoids (**I. b**). CCI induces an increase in thickness and density of the GFAP positive area (**II. b**). High power magnification showed morphological characteristics of GFAP positive cells (astrocytes) and MAP2 positive cells (neurons). Control organoids display astrocytes in resting-state morphology (**I. c**). CCI organoids display astrocytes with characteristics of a reactive phenotype (**II. c**).

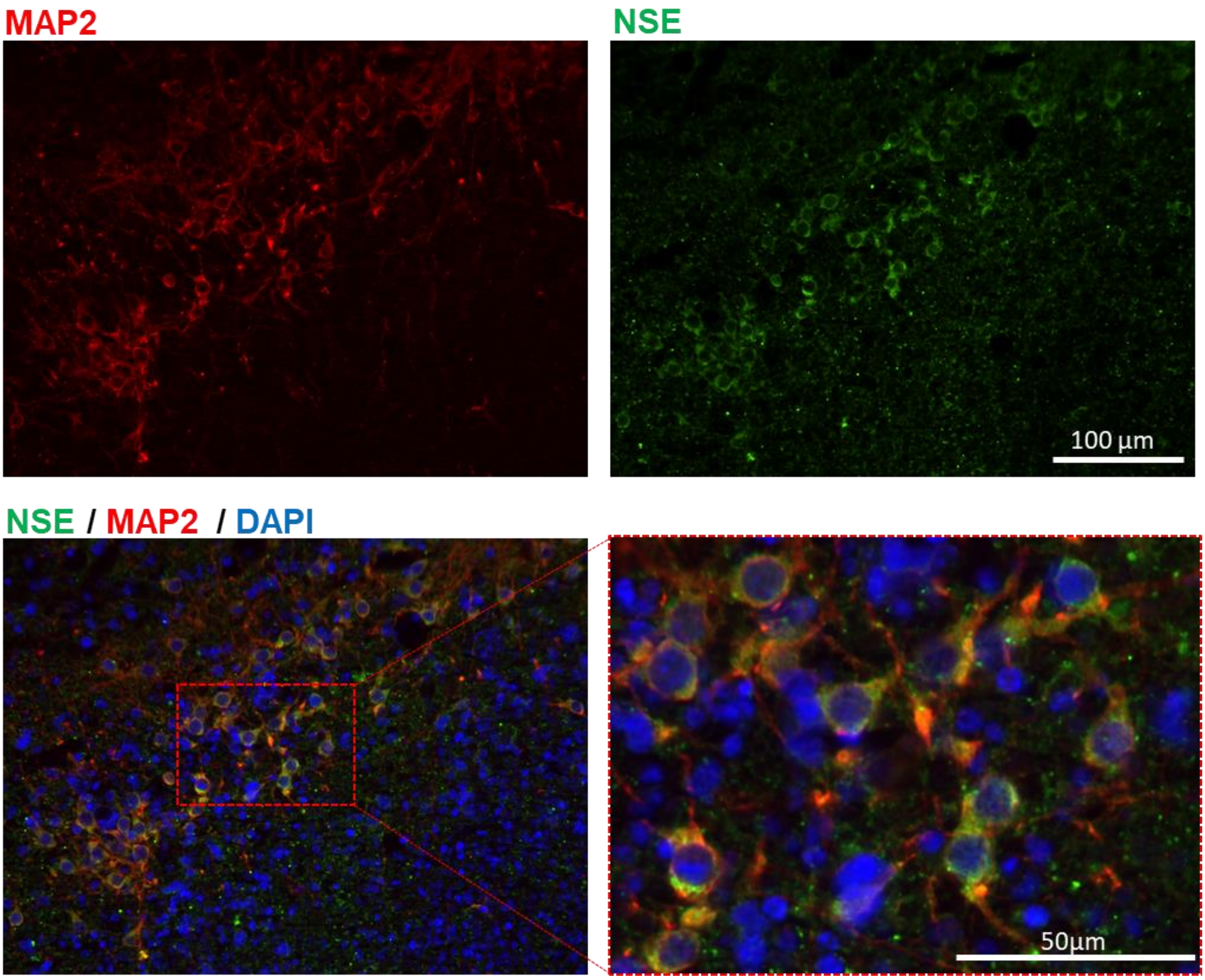

Figure S4. MAP2 and NSE colocalization. MAP2 and NSE immunostainings were performed to corroborate the identity of cells undergoing upregulation of NSE in COs exposed to CCI procedure, microphotographs at 20X show MAP2 and NSE colocalization.

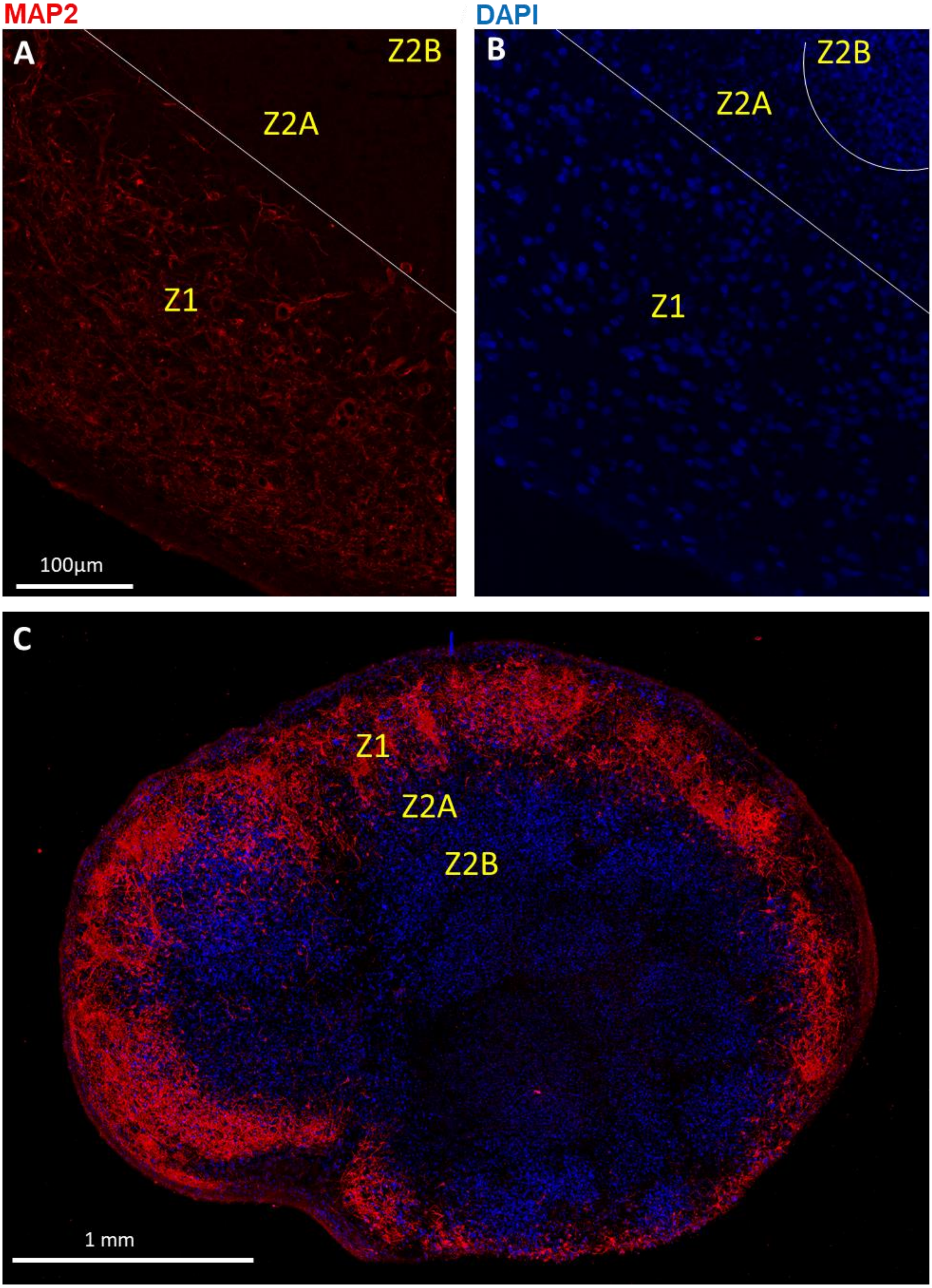

Figure S5. Zonification for analysis of COs**.** MAP2 immunostainings was utilized to determine the area of interest for the analysis performed in COs. Excluded regions due to the absence of MAP2 mark were divided in a transition zone (**A. Z2A**) and the necrotic core (**B. Z2B**) characterized by a higly dense and compacted nucleus by DAPI staining. **C.** Low power magnification of a cerebral organoid showing zonification scheme.
